# Diphlorethohydroxycarmalol Attenuates Fine Particulate Matter-Induced Subcellular Skin Dysfunction

**DOI:** 10.3390/md17020095

**Published:** 2019-02-01

**Authors:** Ao Xuan Zhen, Mei Jing Piao, Yu Jae Hyun, Kyoung Ah Kang, Pincha Devage Sameera Madushan Fernando, Suk Ju Cho, Mee Jung Ahn, Jin Won Hyun

**Affiliations:** 1 School of Medicine, Jeju National University, Jeju 63243, Korea; zhenaoxuan705@gmail.com (A.X.Z.); meijing0219@hotmail.com (M.J.P.); yujae_1113@naver.com (Y.J.H.); legna48@hanmail.net (K.A.K.); sameeramadhu91@gmail.com (P.D.S.M.F.); sukjucho@gmail.com (S.J.C.); 2 Laboratory of Veterinary Anatomy, College of Veterinary Medicine, Jeju National University, Jeju 63243, Korea; healthy@jejunu.ac.kr

**Keywords:** diphlorethohydroxycarmalol, human keratinocytes, PM_2.5_, skin cell damage, MAPK

## Abstract

The skin, the largest organ in humans, is exposed to major sources of outdoor air pollution, such as fine particulate matter with a diameter ≤ 2.5 µm (PM_2.5_). Diphlorethohydroxycarmalol (DPHC), a marine-based compound, possesses multiple activities including antioxidant effect. In the present study, we evaluated the protective effect of DPHC on PM_2.5_-induced skin cell damage and elucidated the underlying mechanisms in vitro and in vivo. The results showed that DPHC blocked PM_2.5_-induced reactive oxygen species generation in human keratinocytes. In addition, DPHC protected cells against PM_2.5_-induced DNA damage, endoplasmic reticulum stress, and autophagy. HR-1 hairless mice exposed to PM_2.5_ showed lipid peroxidation, protein carbonylation, and increased epidermal height, which were inhibited by DPHC. Moreover, PM_2.5_ induced apoptosis and mitogen-activated protein kinase (MAPK) protein expression; however, these changes were attenuated by DPHC. MAPK inhibitors were used to elucidate the molecular mechanisms underlying these actions, and the results demonstrated that MAPK signaling pathway may play a key role in PM_2.5_-induced skin damage.

## 1. Introduction

Particulate matter (PM), which comprises small-sized particles (a diameter of 2.5 to 10 μm), can easily penetrate human exterior organs, such as the eyes, ears, nose, and skin. Among these barriers, the skin is the largest organ on which floating PM can attach [1]. Many studies have reported that PM can even penetrate the skin cells and cause oxidative stress [2]. Consequently, the skin barrier is directly or indirectly damaged, causing skin thickening and wrinkle formation [3]. In addition, when the integrity of the skin is compromised by PM, pathophysiological processes occur in the epidermal keratinocytes and dermal fibroblasts, leading to processes such as apoptosis and aging [4]. Overexposure of cells to PM can trigger the mitogen-activated protein kinase (MAPK) signaling pathway, which is probably related to skin inflammation [5]. Moreover, previous studies have shown that PM analogs can stimulate reactive oxygen species (ROS) generation, causing inflammation, and natural compounds such as resveratrol and curcumin can inhibit ROS production by blocking phosphorylation of MAPKs [6,7].

The edible seaweed *Ishige okamurae* is a brown alga that contains phlorotannins, such as diphlorethohydroxycarmalol (DPHC), and is well known for its abundant bioactive compounds that are used as functional products [8]. Several studies have shown that this marine alga exhibits antitumor, antioxidant, antihypertensive, anticoagulant, anti-inflammatory, antidiabetic, and antibacterial activities [9,10]. We previously reported the cytoprotective effects of DPHC on UVB-induced cell damage in human keratinocytes via inhibition of ROS generation and MAPK signaling [11,12]. The skin barrier was disordered by exposure to PM [2,3,4,5]; however, research on the protective effects of DPHC against PM_2.5_-induced skin damage is rare. In the present study, we aimed to determine the protective effects of DPHC against PM_2.5_-induced skin damage in vitro and in vivo, and to elucidate the underlying mechanisms mediated by the MAPK signaling pathway.

## 2. Results

### 2.1. DPHC Inhibits PM_2.5_-Induced ROS Generation

**Figure 1:**
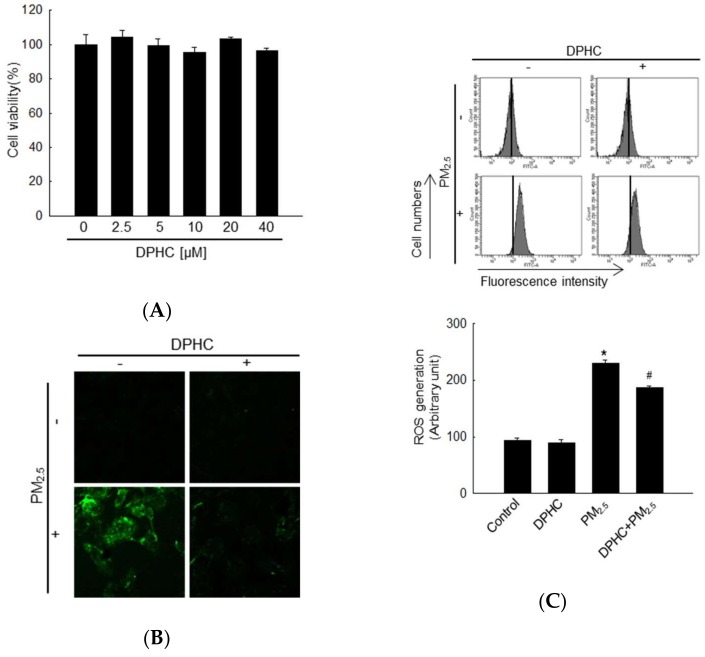
Diphlorethohydroxycarmalol (DPHC) reduced reactive oxygen species (ROS) generation. (**A**) 3-(4,5-dimethylthiazol-2-yl)-2,5-diphenyltetrazolium bromide (MTT) assay was used to determine cell viability after treating HaCaT cells with DPHC (0, 2.5, 5, 10, 20 and 40 µM) for 24 h. ROS generated by PM_2.5_ (fine particulate matter with a diameter ≤ 2.5 µm) were detected using 2′,7′-dichlorodihydrofluorescein diacetate (H_2_DCFDA) staining (25 µM). (**B**) Confocal microscopy and (**C**) flow cytometry were performed to detect intracellular ROS after H_2_DCFDA staining; * *p* < 0.05 and ^#^ *p* < 0.05 compared to control and PM_2.5_-treated groups, respectively.

The results of 3-(4,5-dimethylthiazol-2-yl)-2,5-diphenyltetrazolium bromide (MTT) assay indicate that DPHC showed no cytotoxicity against human keratinocyte cell line, HaCaT cells at all the tested concentrations (0, 2.5, 5, 10, 20, and 40 µM, Figure 1A). We used 20 µM DPHC as the optimal concentration in the subsequent experiments. Confocal microscopic images showed that PM_2.5_-exposed cells exhibited the highest fluorescence intensity with 2′,7′-dichlorodihydrofluorescein diacetate (H_2_DCFDA) staining, which indicates ROS production; however, DPHC inhibited this cellular ROS generation (Figure 1B). Similarly, the blockade of ROS generation by DPHC was confirmed using flow cytometry (Figure 1C). These results showed that DPHC eliminated PM_2.5_-induced ROS generation.

### 2.2. DPHC Inhibits Cellular Macromolecule Damage via Inhibiting PM_2.5_-Induced Oxidative Stress

**Figure 2:**
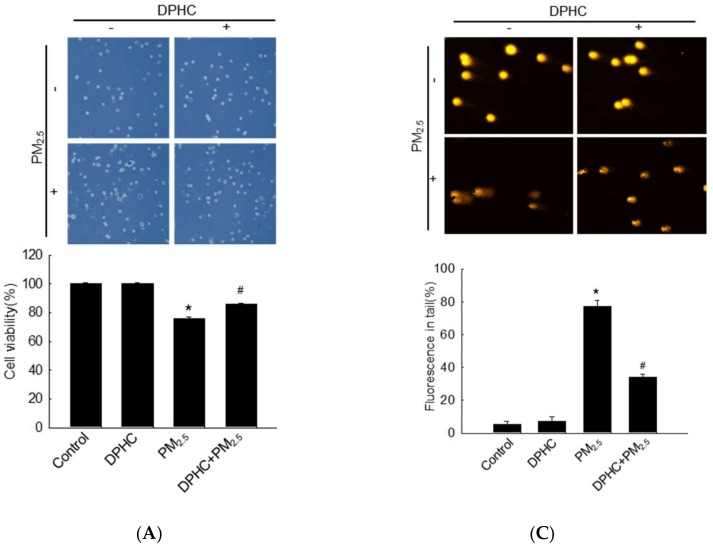

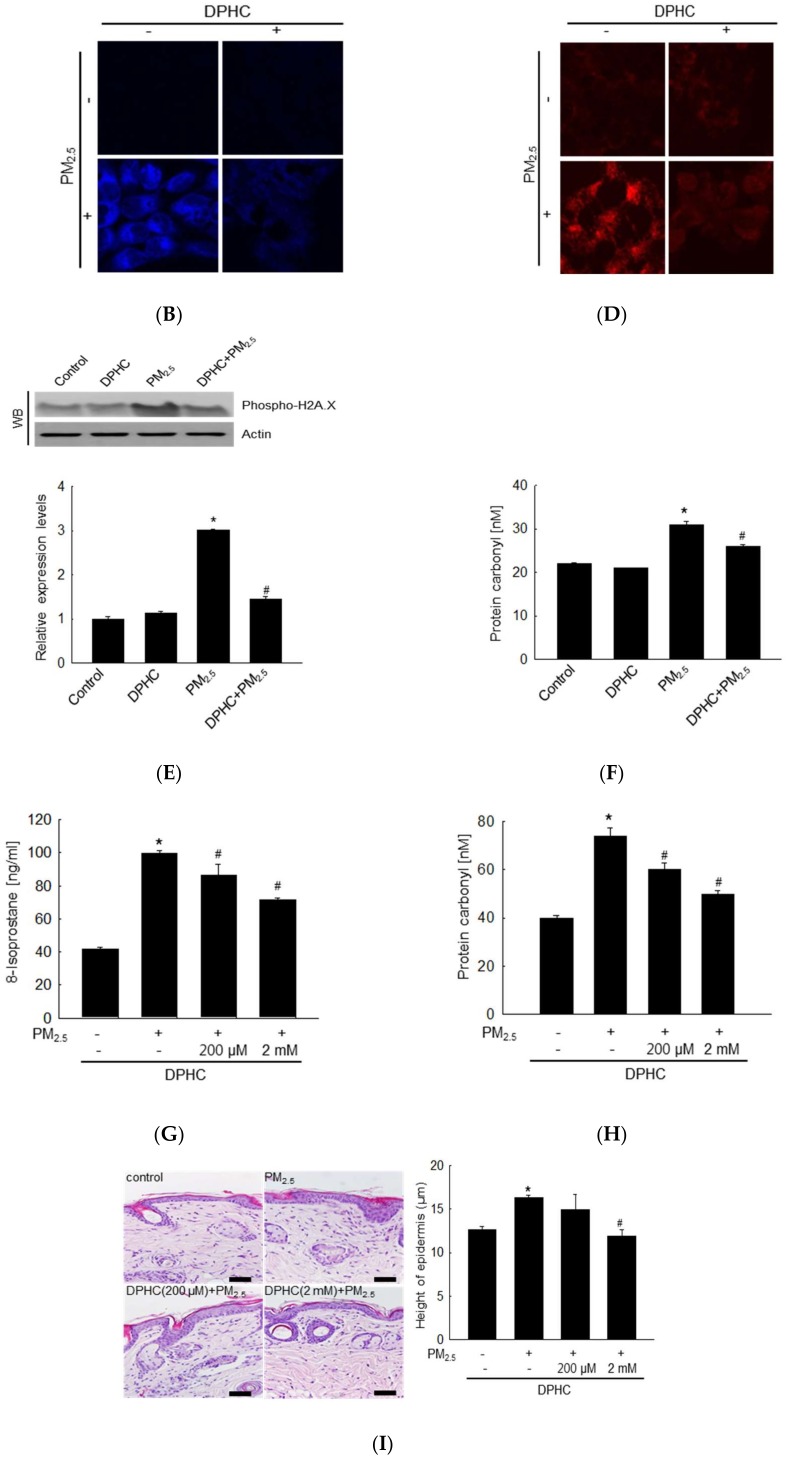
DPHC protected cells against PM_2.5_-induced damage of macromolecules. (**A**) Cell viability was analyzed using trypan blue assay. (**B**) Confocal microscopy was used to analyze lipid peroxidation after diphenyl-1-pyrenylphosphine (DPPP) staining (5 μM). (**C**) Comet assay was used to evaluate DNA damage. (**D**) Avidin-tetramethylrhodamine isothiocyanate (TRITC) (1:200) bound to 8-oxoguanine in DNA was determined using confocal microscopy. (**E**) Cells lysates were analyzed for H2A.X expression using western blotting with actin as loading control. Protein carbonylation was analyzed using a protein carbonyl ELISA kit both (**F**) in vitro and (**H**) in vivo. (**G**) Lipid peroxidation was determined using 8-isoprostaglandin F2α ELISA kit in vivo. (**I**) The mouse epidermal heights were assessed, and images were obtained from every group. Scale bars, 20 μm. * *p* < 0.05 compared to control groups; ^#^ *p* < 0.05 compared to PM_2.5_-treated groups.

The results of trypan blue exclusion assay showed that PM_2.5_ treatment promoted cell death, whereas DPHC reduced the number of dead cells (Figure 2A). Lipid peroxidation caused by PM_2.5_-induced oxidative stress was analyzed using fluorescent diphenyl-1-pyrenylphosphine (DPPP) oxide (Figure 2B). In PM_2.5_-exposed cells, the fluorescence intensity of DPPP oxide was higher than that in cells pretreated with DPHC. DPHC also protected cells against PM_2.5_-induced DNA damage mediated by oxidative stress in the comet assay (Figure 2C). The length of comet tails and percentage of tail fluorescence induced by PM_2.5_ were significantly reduced in cells pretreated with DPHC. Moreover, condensed 8-oxoguanine (8-oxoG) was detected by analyzing binding with avidin-tetramethylrhodamine isothiocyanate (TRITC), and its generation, which was triggered by PM_2.5_, was reduced by DPHC pretreatment (Figure 2D). Additionally, the fragmentation of DNA double strand can trigger the phospho-histone H2A histone family member X (H2A.X). The results were confirmed by using western blotting, which showed that PM_2.5_ treatment induced DNA damage as indicated by the overexpression of phospho-histone H2A.X (Figure 2E). Furthermore, DPHC attenuated protein carbonyl induced by PM_2.5_-induced oxidative stress (Figure 2F). In the in vivo *s*ystem, DPHC inhibited lipid peroxidation (Figure 2G) and protein carbonylation (Figure 2H) induced by PM_2.5_-induced oxidative stress. In addition, PM_2.5_ increased epidermal height, which indicates that PM_2.5_ disordered skin histological architecture. However, DPHC protected the skin against oxidative cellular components, and the epidermal height was lower than that in PM_2.5_-treatment group (Figure 2I). Taken together, these results suggest that DPHC inhibited PM_2.5_-induced oxidative stress both in vitro and in vivo, and protected the skin from PM_2.5_-induced damage.

### 2.3. DPHC Blocks Endoplasmic Reticulum Stress and Autophagy Induced by PM_2.5_

**Figure 3:**
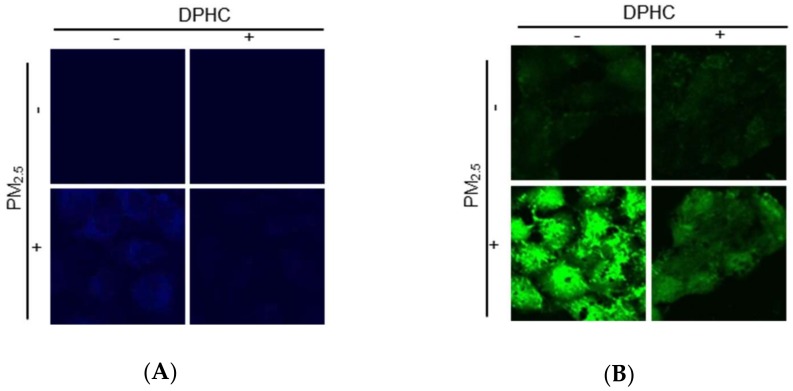

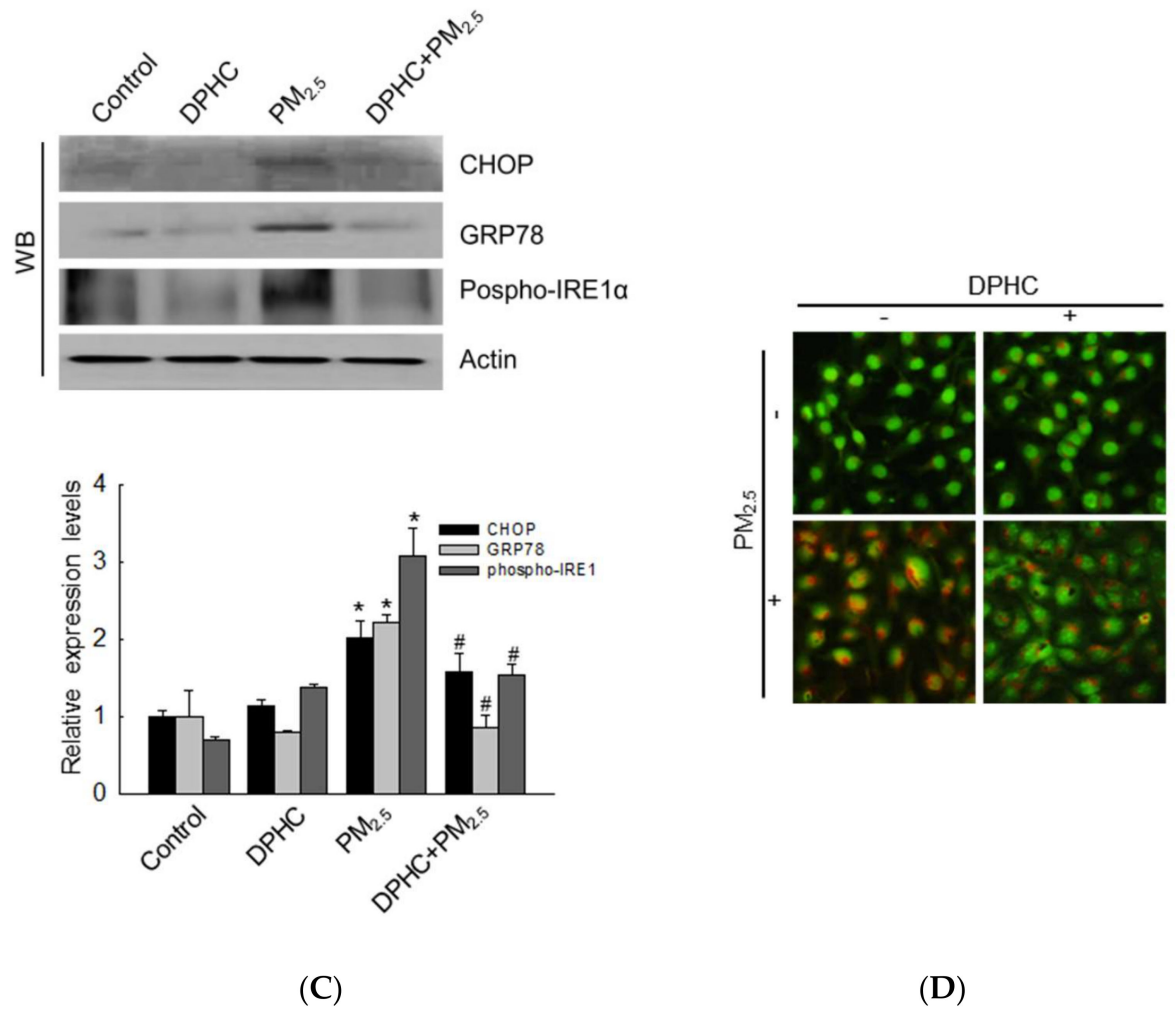

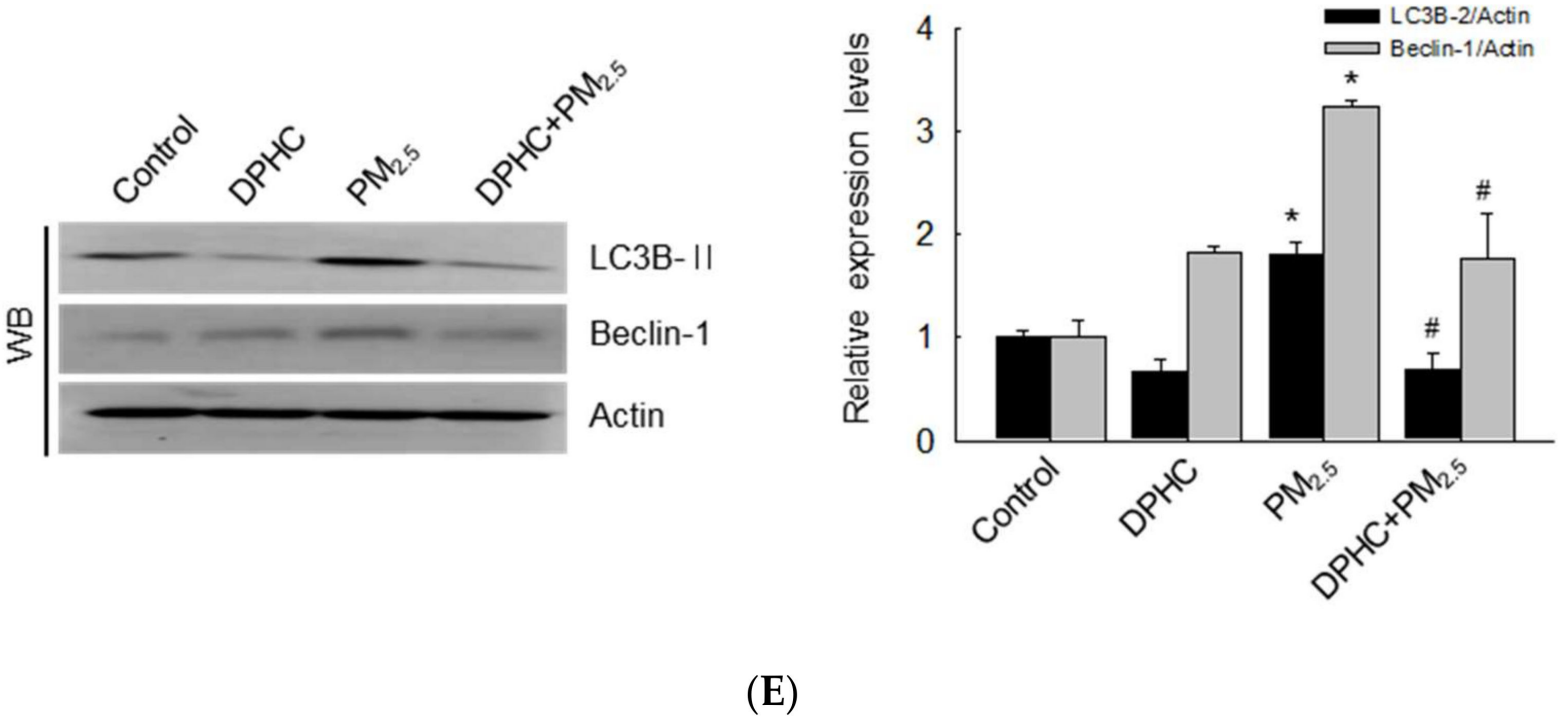
DPHC suppressed PM_2.5_-induced endoplasmic reticulum (ER) stress and autophagy. Cells were stained with (**A**) ER-Tracker Blue-White DPX staining (1 μM) and (**B**) Fluo-4-AM (1 μM) to evaluate ER stress and intracellular Ca^2+^ levels, respectively, using confocal microscopy. (**C**) CCAAT-enhancer-binding protein homologous protein (CHOP), glucose-regulated protein 78 (GRP78), and phospho-inositol-requiring enzyme 1 (IRE1) were detected using western blotting. (**D**) Autophagy was detected using acridine orange (5 μM) with fluorescence microscopy. (**E**) Cell lysates analyzed for protein expression of beclin-1 and LC3B-II using western blotting; * *p* < 0.05 and ^#^ *p* < 0.05 compared to control and PM_2.5_-treated groups, respectively.

Recently, we reported that PM_2.5_-induced oxidative stress resulted in endoplasmic reticulum (ER) stress [13]. ER-Tracker Blue-White DPX is a photostable probe that is selective for the ER and can indicate ER stress. In Figure 3A, PM_2.5_-treated cells showed bright blue color induced by ER stress; however, DPHC attenuated this effect. The ER plays a very important role in Ca^2+^ homeostasis and is the main intracellular Ca^2+^ reservoir [14]. Confocal microscopy was used to analyze the Ca^2+^ level using fluo-4-acetoxymethyl ester (Fluo-4-AM) staining, and PM_2.5_-treated cells exhibited higher fluorescence intensity than the control cells did, which was reduced by DPHC (Figure 3B). ER stress induces CCAAT-enhancer-binding protein homologous protein (CHOP), which is involved in apoptosis [15]. Furthermore, under the pressure of ER stress, the glucose-regulated protein 78 (GRP78) activates serine/threonine-protein kinase/endoribonuclease inositol-requiring enzyme 1 (IRE1)-α, protein kinase RNA-like endoplasmic reticulum kinase (PERK), and activating transcription factor 6 (ATF6) signaling pathways, which ultimately promote cell apoptosis [16]. As shown in Figure 3C, ER stress-related proteins such as CHOP, GRP78, and phospho-IRE1 were increased by PM_2.5_-treatment. However, DPHC decreased the levels of these three proteins. These findings suggested that PM_2.5_-induced ER stress was inhibited by DPHC pretreatment.

ER stress is also related to autophagy, which is a self-degradative process [17]. Therefore, we also determined whether PM_2.5_ promotes autophagy. The intracellular acidity caused autophagic lysosomes to exhibit orange/red fluorescent cytoplasmic vesicles, while the nuclei appeared green. In PM_2.5_-treated cells stained by acridine orange, which is the lysosome marker dye, intracellular vacuoles accumulated dramatically, but this effect was inhibited by DPHC (Figure 3D). In addition, PM_2.5_ upregulated the protein levels of beclin-1 and light chain 3B (LC3B)-II, which participate in initiating autophagosome formation during autophagy and the processed form of LC3, respectively. However, the expression levels of these two proteins were reduced by DPHC (Figure 3E). These results suggested that DPHC suppressed PM_2.5_-induced autophagy in skin cells.

### 2.4. PM_2.5_-Induced Mitochondrial Damage is Weakened by DPHC

**Figure 4:**
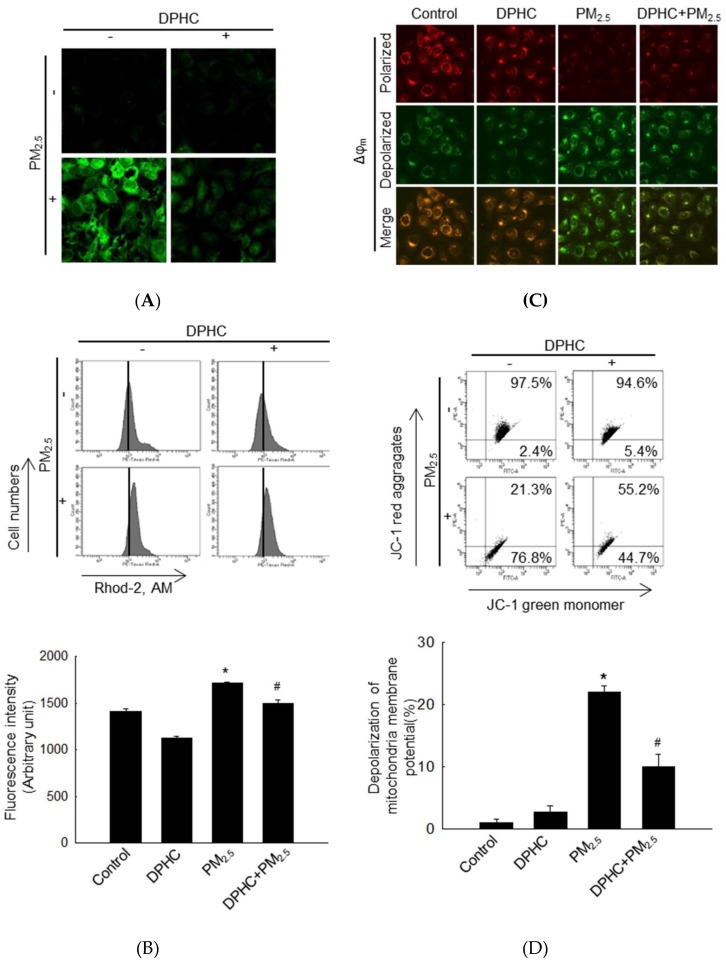

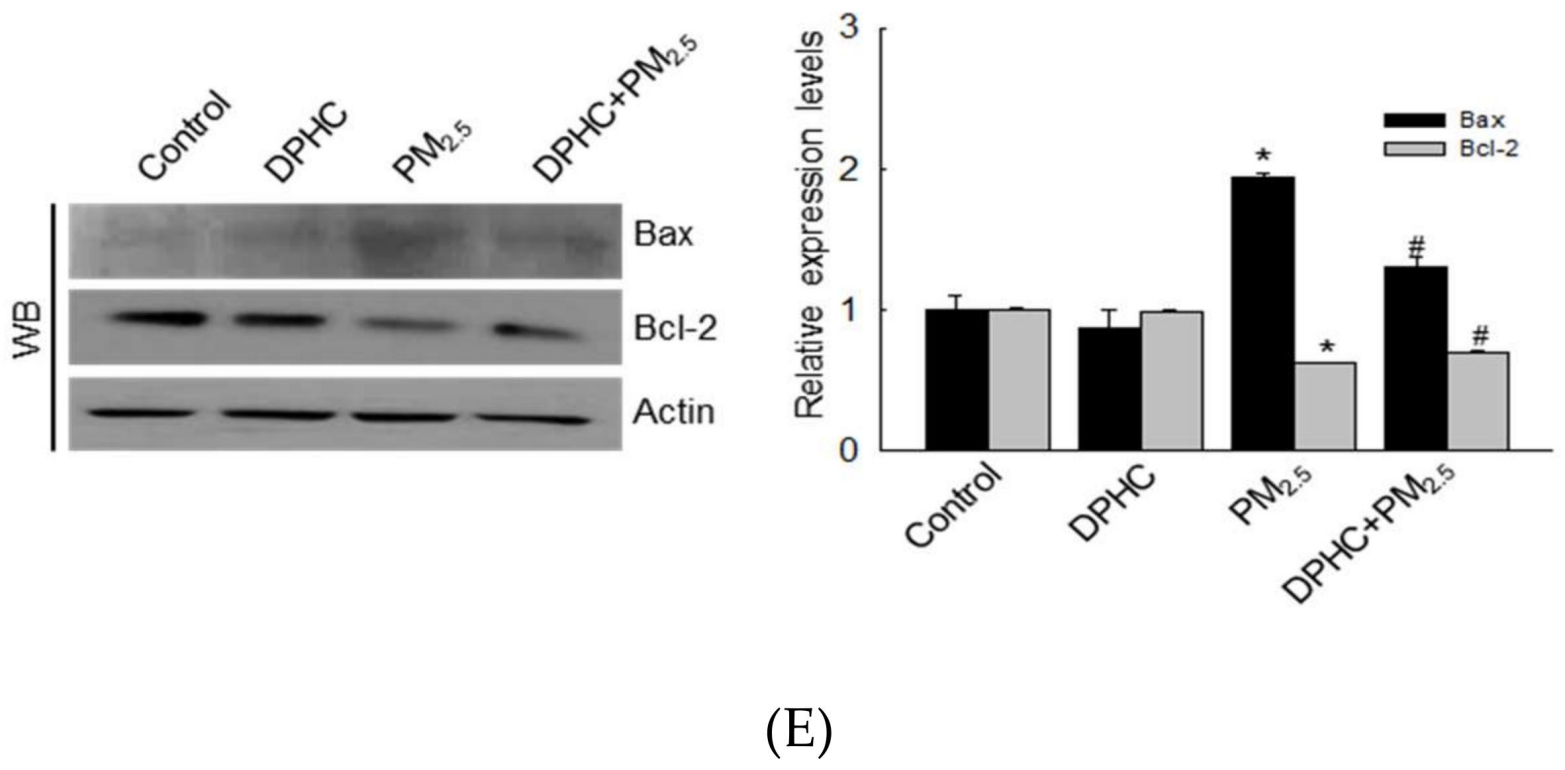
PM_2.5_-induced mitochondrial damage was blocked by DPHC. (**A**) Mitochondrial ROS was assessed using confocal microscopy after staining with dihydrorhodamine 123 (DHR123) (10 μM). (**B**) Mitochondrial Ca^2+^ levels were analyzed by Rhod-2 AM staining (1 μM) using flow cytometry. (**C**, **D**) Mitochondrial membrane potential (Δψ_m_) was detected by JC-1 staining (2 μM) using confocal microscopy and flow cytometry, separately. (E) Cell lysates were analyzed for Bax and Bcl-2 protein expression using western blotting; * *p* < 0.05 and ^#^ *p* < 0.05 compared to control and PM_2.5_-treated groups, respectively.

Mitochondrial membrane permeability is related to apoptosis through activation of caspase-associated proteins [18]. Dihydrorhodamine 123 (DHR123), a mitochondria ROS indicator, was used for the measurement of mitochondrial oxidative stress, and the result demonstrated that overexpression of mitochondrial ROS was induced by PM_2.5_ but reduced by DPHC (Figure 4A). Similarly, mitochondrial Ca^2+^ levels were increased by PM_2.5_ but decreased by DPHC in the rhod-2 acetoxymethyl ester (Rhod-2 AM) staining assay (Figure 4B). JC-1 dye was used to detect mitochondrial membrane potential (Δψ_m_), and the red and green fluorescence indicate membrane polarization and depolarization, respectively. Furthermore, the results showed that Δψ_m_ depolarization was increased by PM_2.5_ but was suppressed by DPHC as shown in the confocal microscopy and flow cytometry analyses (Figure 4C,D). In addition, the B-cell lymphoma-2 (Bcl-2) family members control mitochondrial permeability to regulate apoptosis. Therefore, we examined the expression levels of the pro-apoptotic Bcl-associated X protein (Bax) and the anti-apoptotic Bcl-2 proteins. The results illustrated that PM_2.5_ increased Bax levels and decreased Bcl-2 levels in keratinocytes, which were reversed by DPHC pretreatment (Figure 4E). Therefore, DPHC suppressed PM_2.5_-induced mitochondrial ROS generation and balanced the membrane permeability.

### 2.5. PM_2.5_ Accelerates Apoptotic Cell Death

**Figure 5:**
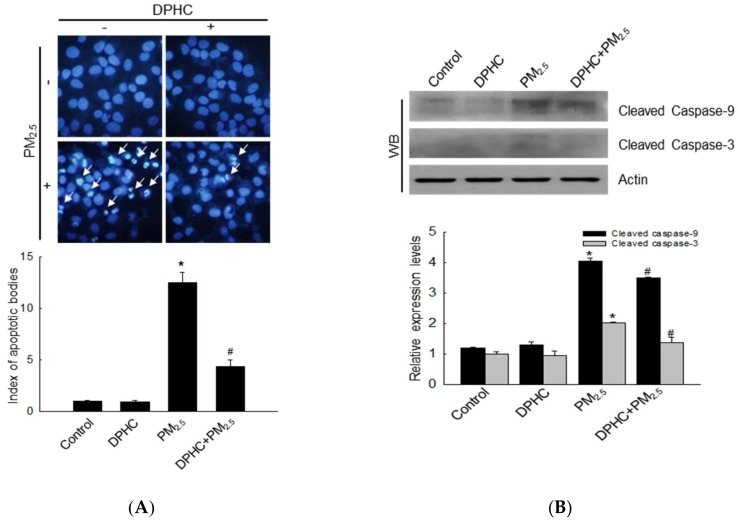

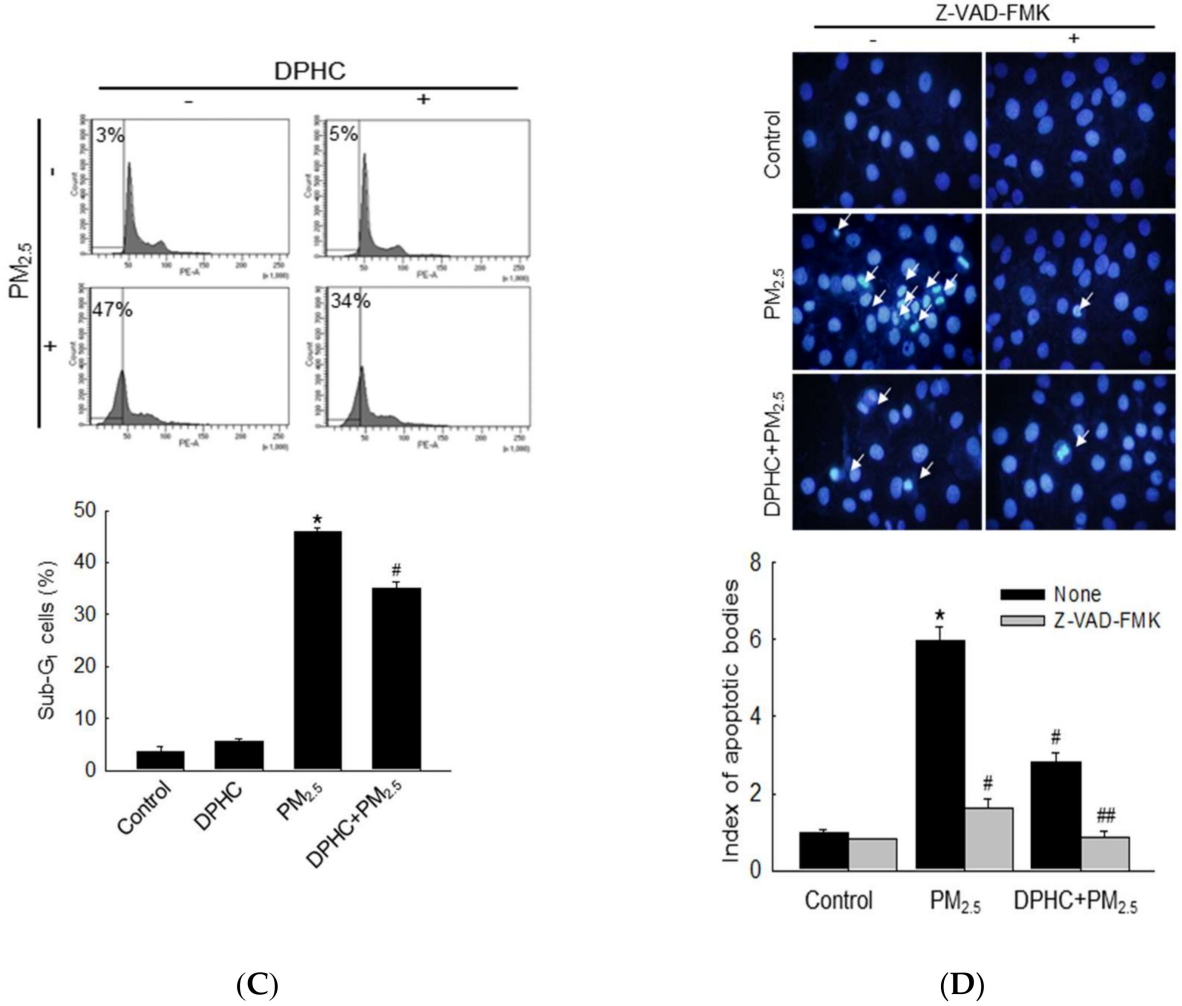
DPHC inhibited PM_2.5_-induced cell apoptosis. (**A**, **D**) Cells were pretreated with DPHC, pan caspase inhibitor, Z-VAD-FMK (30 μM), or both for 1 h, followed by PM_2.5_ treatment for another 24 h. Apoptotic body formation was detected using Hoechst 33342 staining (20 μM); apoptotic bodies are indicated by arrows. (**B**) Cell lysates were analyzed for protein levels of caspase-9 and caspase-3 using western blotting. (**C**) Sub-G_1_ cells were counted using flow cytometry with PI staining; * *p* < 0.05, ^#^ *p* < 0.05, and ^##^ *p* < 0.05 compared with control, PM_2.5_-exposed, and PM_2.5_-exposed plus DPHC-pretreated cells, respectively.

Hoechst 33342 staining is used to visualize condensed nuclei in apoptotic cells and PM_2.5_ induced accumulation of apoptotic bodies, whereas DPHC pre-treatment diminished these effects (Figure 5A). Additional evidences showed that PM_2.5_ promoted the expression of cleaved caspase-9 and caspase-3 (Figure 5B), which suggested that caspase activation may be involved in cell apoptosis. The effect was attenuated by DPHC. In addition, PM_2.5_ significantly increased the sub-G_1_ phase cells compared with that in the control group, and this effect was inhibited by DPHC (Figure 5C). To confirm the effect of caspase activation on apoptosis, we used the irreversible caspase inhibitor, Z-VAD-FMK. Notably, Z-VAD-FMK, DPHC, or both decreased the number of apoptotic cells (Figure 5D). Thus, these results indicated that DPHC exhibits cytoprotective effects on PM_2.5_-induced apoptosis.

### 2.6. DPHC Regulates PM_2.5_-Induced Apoptosis via MAPK Signaling Pathways

**Figure 6:**
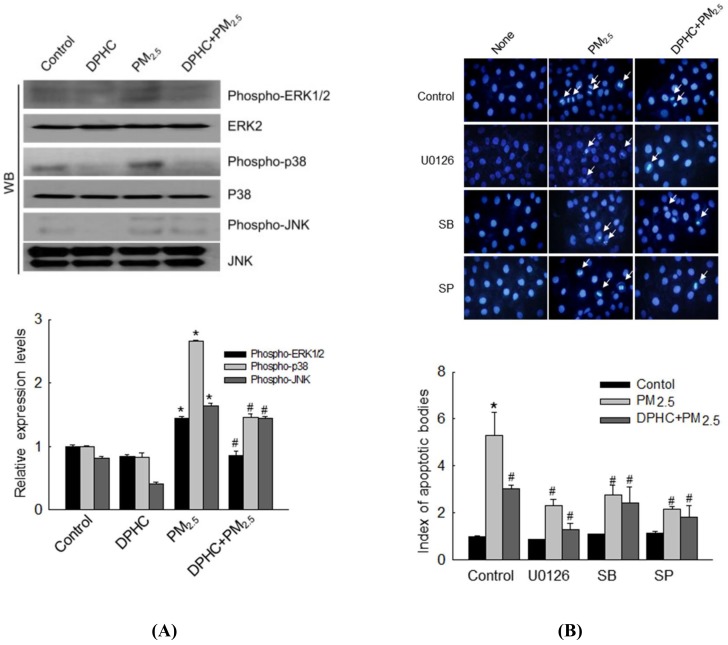
DPHC prevented PM_2.5_-induced cell apoptosis via mitogen-activated protein kinase (MAPK) signaling pathway. (**A**) Cell lysates were analyzed to detect phosphorylation of ERK, p38 MAPK, and c-Jun *N*-terminal kinase (JNK) using western blotting. (**B**) Analysis of Hoechst 33342-stained apoptotic cells after treatment with U0126 (50 nM), SB203580 (SB, 10 μM), and SP600125 (SP, 5 μM), which are inhibitors of ERK, p38 MAPK, and JNK, respectively; * *p* < 0.05 and ^#^ *p* < 0.05 compared with control and PM_2.5_-exposed cells, respectively.

To further explore the potential involvement of MAPK signaling pathways in PM_2.5_-mediated modulation of apoptosis, MAPK-related proteins, extracellular signal-regulated kinase (ERK), p38, and c-Jun *N*-terminal kinase (JNK) were detected using western blotting. The results showed that PM_2.5_ increased the formation of phospho-ERK1/2, phospho-p38, and phospho-JNK compared to the levels in control cells. However, phosphorylation of these proteins was reversed by DPHC pretreatment (Figure 6A). In addition, after pretreatment of cells with U0126, SB203580, and SP600125 (inhibitors of ERK, p38, and JNK, respectively), apoptotic bodies were detected using Hoechst 33342 staining (Figure 6B). The results showed that all the inhibitors decreased PM_2.5_-induced apoptotic bodies, similar to cells pretreated with DPHC. Therefore, DPHC inhibited MAPK signaling pathway and prevented PM_2.5_-induced apoptosis.

## 3. Discussion

Previous studies showed that DPHC suppressed ROS generation by blocking matrix metalloproteinase (MMP)-1 expression [12] and activating the nucleotide excision repair system to inhibit UVB-induced DNA damage in human HaCaT cells [19]. DPHC exhibits antioxidant [8,20], antiviral [21], hypoglycemic [22], and anti-melanogenesis effects [23], as well as protective action against gamma ray radiation [24]. Studies have also shown that PM_2.5_ may penetrate the skin barrier and damage the keratinocytes [25,26]. We tested the protective effects of DPHC on PM_2.5_-induced skin damage in this study. DPHC, as a phlorotannin, showed no toxicity to human HaCaT cells from concentrations ranging from 2.5 to 40 µM. In addition, our previous studies showed that DPHC inhibited both superoxide anions and hydroxyl radicals directly at 20 µM [11]. Therefore, in the subsequent experiments, we used DPHC at 20 µM as the test concentration. A recent study demonstrated that PM_2.5_ changed the morphology of keratinocytes because of ROS overproduction, which damaged the intracellular antioxidant system [27]. In our recent study [13], we conducted experiments with 25, 50, 75, and 100 µg/mL of PM_2.5_ for 24 h in cells and 50, 100, 200, and 400 µg/mL of PM_2.5_ for 7 consecutive days in animals. Because 50 µg/mL in vitro and 100 µg/mL in vivo showed skin damage, we evaluated the effect of PM_2.5_ on skin damage at 50 µg/mL in cells and at 100 µg/mL in animal model. We investigated PM_2.5_-induced intracellular ROS production in keratinocytes and the results showed that PM_2.5_ promoted ROS generation, lowered cell viability, and damaged cell structures such as DNA, and induced lipid peroxidation. However, DPHC blocked intracellular ROS generation, increased cell viability, and protected the cell structure. In vivo experiments also proved that polyunsaturated fatty acid oxidation and protein carbonylation as well as epidermal height were increased by PM_2.5_; nevertheless, the generation of these two substances and increase in epidermal height were inhibited by DPHC.

ER stress plays a crucial role in intracellular dysfunction and may be induced by PM_2.5_ [15]. In ER transmembrane protein regulation, GRP78 facilitates misfolded protein refolding of mainly PERK, IRE-1α, and ATF6 [28]. These results showed that DPHC suppressed PM_2.5_-induced ER stress and balanced Ca^2+^ dynamics, which is essential to ER function. In addition, PM_2.5_ induced the phosphorylation of IRE-1 and upregulated protein levels of GRP78 and CHOP, which indicated that PM_2.5_ activated the ER stress pathway in HaCaT cells. Furthermore, these effects were reversed by DPHC. A previous review reported that ER stress may induce cell death through autophagy [29]. In the autophagic process, various autophagy-related genes (ATGs) including ATG5, beclin-1, and the microtubule-associated protein LC3B play a very important role. Beclin-1 is involved in nucleation of phagophore and formation of autophagosomes [30]. Therefore, we detected the protein levels of beclin-1 and LC3- II, which were increased by PM_2.5_; however, DPHC decreased this effect.

In addition to ER stress, mitochondrial dysfunction is related to intracellular ROS generation and causes cell damage. Previous studies proved that elevated ROS levels induced by UVA irradiation also decrease the Δψ_m_, which induces the generation of cytochrome c and apoptosis-related factors [31]. Our results demonstrated that PM_2.5_ not only induced mitochondrial ROS generation, but also caused mitochondrial swelling. In addition, PM_2.5_ promoted the protein levels of Bax, which is an apoptosis-related protein, and blocked the level of Bcl-2, an anti-apoptotic protein. However, DPHC reversed all these effects, suggesting that it protected cells against PM_2.5_-induced mitochondrial damage.

Because PM2.5 may be related to cell apoptosis, we examined nuclear condensation, which is one of the characteristics of apoptotic cells. The result showed that PM2.5 promoted the development of apoptotic bodies and activated caspases-3 and 9, two key apoptotic proteins. However, DPHC inhibited cell apoptosis, which suggested that it protected the cells from apoptosis by regulating the levels of apoptosis-associated proteins.

Previously, p38 MAPK was shown to degrade Bcl-2 and activate Bax, resulting in mitochondrial-mediated apoptotic cell death [32,33,34]. Therefore, we detected the expression levels of MAPK signaling-associated proteins. Phosphorylation of ERK p38 and JNK was upregulated by PM2.5 and downregulated by DPHC pretreatment. The effects of MAPK signaling were further explored using ERK, p38, and JNK inhibitors. DPHC pretreatment reduced apoptotic cell number. MAPK signaling-related inhibitors also contributed to reducing the number of apoptotic bodies, which indicated that DPHC may inhibit cell death through the MAPK signaling pathway.

## 4. Materials and Methods

### 4.1. PM_2.5_ Preparation

There have been reports that PM_2.5_ in Korea mainly originates from incomplete coal combustion and diesel vehicle exhausts [35,36]. Therefore, we used PM_2.5_, which is a standard diesel PM (SRM 1650b) issued by the National Institute of Standards and Technology (NIST, Gaithersburg, MD, USA). It was purchased from Sigma-Aldrich (St. Louis, MO, USA). The 1650b diesel PM was mainly composed of polycyclic aromatic hydrocarbons (PAHs) and nitro-PAHs. PM_2.5_ was dispersed in dimethyl sulfoxide (DMSO) to obtain a 25 mg/mL stock solution, which was sonicated for 30 min to avoid agglomeration of the suspended PM_2.5_ particles. Experiments were performed with the stock solution within 1 h to avoid variability in PM_2.5_ composition in solution. In real-world settings, the human skin is exposed to airborne particulate matter. Considering the limitations of laboratory research, the cells were exposed to solutions of PM_2.5_. In our recent report, PM_2.5_ induced morphological damage at 50 μg/mL in cell system and at 100 μg/mL in the animal system [13]. Therefore, we used 50 μg/mL of PM_2.5_ in cell system and 100 μg/mL in animal system for this study.

### 4.2. Cell Culture

The human keratinocyte cell line HaCaT was obtained from Cell Lines Service (Heidelberg, Germany) and cultured at 37 °C in a 5% CO_2_ incubator exposed to a humidified atmosphere. The cells were maintained in Dulbecco’s modified Eagle’s medium (DMEM) with 10% heat-inactivated fetal bovine serum and antibiotic–antimycotic solution (100 units/mL penicillin, 100 µg/mL streptomycin, and 0.25 µg/mL amphotericin B, Gibco, Life Technologies Co., Grand Island, NY, USA).

### 4.3. Cell Viability

MTT assay was used to assess the cytotoxicity of DPHC on HaCaT cells. Cells (1.0 × 10^5^ cells/well) were plated on a 24-well plate, incubated for 16 h, and exposed to 2.5, 5, 10, 20, or 40 µM DPHC. The MTT stock solution (2 mg/mL) was added, and the cells were incubated for another 4 h to yield formazan crystals. After dissolving the crystals in DMSO, the absorbance was detected at 540 nm using a scanning multi-well spectrophotometer.

### 4.4. Animal Experiments

HR-1 hairless male mice (OrientBio, Kyungki-do, Republic of Korea) were used for the in vivo experiments under the guidelines for the care and use of laboratory animals at Jeju National University (Jeju, Republic of Korea; permit number: 2017-0026). The mice were randomly divided into the following three groups (*n* = 4 each): control; PM_2.5_-treated; and DPHC (200 µM) + PM_2.5_-treated or DPHC (2 mM) + PM_2.5_-treated groups. Skin patches were created on the dorsal part for sampling to analyze skin damage-related molecules. PM_2.5_ was prepared at a concentration of 100 μg/mL, loaded on propylene glycol, and then spread on a nonwoven polyethylene pad over a 1 cm^2^ area. Then, the mouse dorsal skin was placed in continuous contact with the pads for 7 days, followed by immediate dissection of the treated tissue for histological and biochemical analyses.

### 4.5. Determination of ROS

H_2_DCFDA was used to detect the levels of intracellular ROS and DHR123 was used for mitochondrial ROS [13]. Cells were seeded for 16 h and then treated with 20 µM DPHC, PM_2.5_ (50 µg/mL), or both at 37 °C for another 24 h. The data were obtained after staining the cells with H_2_DCFDA (Molecular Probes, Eugene, OR, USA) and DHR123 (Molecular Probes) for 30 min at 37 °C. A laser scanning confocal microscope with the FV10-ASW viewer 4.2 software (Carl Zeiss, Oberkochen, Germany) was use for imaging analysis, while the stained cells were counted using a high-performance flow cytometer.

### 4.6. Trypan Blue Staining

To detect the cytoprotective effect of DPHC against PM_2.5_-induced cell damage, we used trypan blue exclusion assay, which cannot stain live cells with intact cell membranes [13]. Cells were seeded and then treated with DPHC, PM_2.5,_ or both for 24 h. Then, the cell suspension was incubated with 5 µL 0.1% trypan blue solution for 5 min at room temperature and a light microscope was used to count the number of viable and dead cells.

### 4.7. Lipid Peroxidation Assay

Lipid peroxidation was detected by reacting with the fluorescent probe DPPP (Molecular Probes). Then, images of the cellular fluorescent product were captured using confocal microscope.

### 4.8. Comet Assay

DNA strand breakage was determined using alkaline single cell gel electrophoresis [37]. Harvested cells were mounted on slides, coated with agarose gel, and immersed in lysis buffer. After staining with ethidium bromide, the slides were observed using a fluorescence microscope equipped with the image analysis software (Kinetic Imaging, Komet 5.5, Liverpool, UK). Images of damaged and undamaged DNA were captured as comet tail and head, respectively. The percentage of total fluorescence in the comet tail and the tail length of 50 cells per slide were recorded.

### 4.9. Detection of 8-Oxoguanine

To detect the oxidative modification of bases in DNA, 8-oxoG level was detected [13]. Cells were fixed in the chamber slides, and the 8-oxoG level was estimated by staining with avidin-TRITC conjugate (Sigma-Aldrich). Images were obtained using confocal microscope.

### 4.10. Western Blotting

Cell lysates were separated using sodium dodecyl sulfate-polyacrylamide gel electrophoresis (SDS-PAGE), and the separated proteins were transferred onto pure nitrocellulose blotting membrane (Pall Gelman Laboratory, Ann Arbor, MI, USA). The membranes were incubated with primary antibodies against phospho-H2A.X (Ser139), CHOP, beclin-1, LC3B, caspase-3, caspase-9, ERK, JNK (Cell Signaling Technology, Beverly, MA, USA), GRP78, Bax, phospho-p38 (Santa Cruz Biotechnology, Santa Cruz, CA, USA), phospho-IRE1 (Abcam, Cambridge, MA, USA), and actin (Sigma-Aldrich), followed by incubating with a corresponding secondary antibody (Pierce, Rockford, IL, USA). The Amersham enhanced chemiluminescence plus western blotting detection system (GE Healthcare Life Sciences, Buckinghamshire, UK) was used to detect the protein bands.

### 4.11. Protein Carbonylation Assay

Protein carbonyl level was measured to detect the oxidative modification of proteins [37]. Cells and mouse skin lysates were analyzed to detect protein oxidation using an Oxiselect^TM^ protein carbonyl enzyme-linked immunosorbent assay (ELISA) kit (Cell Biolabs, San Diego, CA, USA), according to the manufacturer’s instructions.

### 4.12. 8-Isoprostane Assay

To detect the oxidative modification of lipids, 8-isoprostane level was measured [38]. The levels of 8-isoprostane in the mouse skin tissue was assayed using the Oxiselect^TM^ 8-iso-prostaglandin F2α ELISA kit (Cell Biolabs, San Diego, CA, USA), according to the manufacturer’s instructions.

### 4.13. ER Staining

The fluorescent images of the ER were acquired using confocal microscope after reacting with an ER-tracker blue-white DPX dye (Molecular Probes) [39].

### 4.14. Quantification of Ca^2+^ Level

Cells were stained with Fluo-4-AM or Rhod-2 AM (Molecular Probes) to detect intracellular or mitochondrial Ca^2+^ respectively. The Fluo-4-AM fluorescence was assessed using confocal microscopy and Rhod-2 AM was detected using flow cytometer.

### 4.15. Mitochondrial Δψ Membrane Potential (Δψ_m_) Analysis

Δ*ψ_m_* was analyzed by staining with 5,5′,6,6′-tetrachloro-1,1′,3,3′-tetraethylbenzimidazolylcarbocyanine iodide (JC-1, Invitrogen, Carlsbad, CA, USA), a lipophilic cationic fluorescence dye [40]. After pretreatment with DPHC, the cells were exposed to PM_2.5_ for another 5 h. Then, the cells were stained with JC-1, and analyzed using confocal microscopy and flow cytometry.

### 4.16. Acridine Orange Morphology Assay

To analyze autophagy, the cells were reacted with acridine orange (Invitrogen) for 15 min and fluorescence was measured using a fluorescence microscope (BH2-RFL-T3; Olympus, Tokyo, Japan). Depending on acidity, autophagic lysosomes appeared as orange/red fluorescent cytoplasmic vesicles, while the nuclei were stained green [27].

### 4.17. Hoechst 33342 Staining

To detect the apoptotic body, the DNA-specific fluorescent dye Hoechst 33342 was used [40]. Cells were pre-treated with 20 µM DPHC, the inhibitors, or both for 1 h, followed by PM_2.5_ for another 24 h. The stimulated cells were stained with Hoechst 33342 (Sigma-Aldrich) and visualized using a fluorescence microscope. Then, the images of proportions of the apoptotic cells were acquired using a CoolSNAP-Pro color digital camera (Media Cybernetics, Rockville, MD, USA).

### 4.18. Detection of Sub-G_1_-Phase Cells

To determine the number of apoptotic cells, sub-G_1_ hypo-diploid cells were assessed using flow cytometric analysis [24]. Harvested cells were immersed in 70% ethanol at 4 °C for 30 min and then they were incubated in a mixture of 50 mg/mL propidium iodide (PI) and 50 μg/mL RNase A in the dark at 37 °C for another 30 min. Finally, the cells were counted using a FACSCalibur flow cytometer (Becton Dickinson, Franklin Lakes, NJ, USA).

### 4.19. Statistical Analysis

The statistical significance among the different groups was analyzed using the Tukey’s tests with the SigmaStat version 3.5 software (Systat Software Inc., San Jose, CA, USA). All the data were expressed as the means ± standard error, and a *p* < 0.05 was considered statistically significant.

## 5. Conclusions

**Figure 7:**
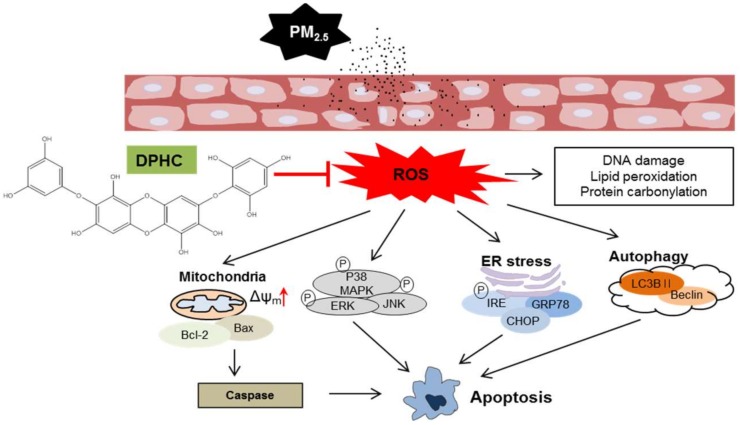
Schematic diagram showing the protective mechanism of DPHC on PM_2.5_-induced skin damage. Skin exposed to PM_2.5_ generates excessive reactive oxygen species (ROS) production, which leads to mitochondrial dysfunction, endoplasmic reticulum (ER) stress, autophagy, and even apoptosis. However, DPHC triggers cytoprotective mechanism by blocking oxidative stress cellular components and inhibiting MAPK signaling apoptotic pathway.

In conclusion, our study showed that PM_2.5_ accelerated skin cell death by generating ROS, damaging complex macromolecules, and destroying the structure of cellular organelles (Figure 7). During this process, ER stress, mitochondrial depolarization, and lysosome generation were promoted, but DPHC protected the skin cells against PM_2.5_-induced damage through the MAPK signaling pathway. All these results enhance the understanding of the mechanisms underlying PM_2.5_-induced destruction of the skin, and demonstrate the potential usefulness of DPHC in protecting the skin from PM_2.5_ exposure.

## References

[1] Huang P.H., Tseng C.H., Lin C.Y., Lee C.W., Yen F.L. (2018). Preparation, characterizations and anti-pollutant activity of 7,3′,4′-trihydroxyisoflavone nanoparticles in particulate matter-induced HaCaT keratinocytes. Int. J. Nanomed..

[2] Puri P., Nandar S.K., Kathuria S., Ramesh V. (2017). Effects of air pollution on the skin: A review. Indian J. Dermatol. Venereol. Leprol..

[3] Kim K.E., Cho D., Park H.J. (2016). Air pollution and skin diseases: Adverse effects of airborne particulate matter on various skin diseases. Life Sci..

[4] Morita A. (2007). Tobacco smoke causes premature skin aging. J. Dermatol. Sci..

[5] Bosch R., Philips N., Suárez-Pérez J.A., Juarranz A., Devmurari A., Chalensouk-Khaosaat J., González S. (2015). Mechanisms of photoaging and cutaneous photocarcinogenesis, and photoprotective strategies with phytochemicals. Antioxidants.

[6] Tsai M.H., Hsu L.F., Lee C.W., Chiang Y.C., Lee M.H., How J.M., Wu C.M., Huang C.L., Lee I.T. (2017). Resveratrol inhibits urban particulate matter-induced COX-2/PGE2 release in human fibroblast-like synoviocytes via the inhibition of activation of NADPH oxidase/ROS/NF-κB. Int. J. Biochem. Cell Biol..

[7] Rennolds J., Malireddy S., Hassan F., Tridandapani S., Parinandi N., Boyaka P.N., Cormet-Boyaka E. (2012). Curcumin regulates airway epithelial cell cytokine responses to the pollutant cadmium. Biochem. Biophys. Res. Commun..

[8] Heo S.J., Kim J.P., Jung W.K., Lee N.H., Kang H.S., Jun E.M., Park S.H., Kang S.M., Lee Y.J., Park P.J. (2008). Identification of chemical structure and free radical scavenging activity of diphlorethohydroxycarmalol isolated from a brown alga, ishige okamurae. J. Microbiol. Biotechnol..

[9] Kang N.J., Han S.C., Kan G.J., Koo D.H., Koh Y.S., Hyun J.W., Lee N.H., Ko M.H., Kang H.K., Yoo E.S. (2015). Diphlorethohydroxycarmalol inhibits interleukin-6 production by regulating NF-kB, stat5 and socs1 in lipopolysaccharide-stimulated raw 264.7 cells. Mar. Drugs.

[10] Mayer A.M., Hamann M.T. (2005). Marine pharmacology in 2001-2002: Marine compounds with anthelmintic, antibacterial, anticoagulant, antidiabetic, antifungal, anti-inflammatory, antimalarial, antiplatelet, antiprotozoal, antituberculosis, and antiviral activities; affecting the cardiovascular, immune and nervous systems and other miscellaneous mechanisms of action. Comp. Biochem. Physiol. C Toxicol. Pharmacol..

[11] Piao M.J., Kang K.A., Kim K.C., Chae S., Kim G.O., Shin T., Kim H.S., Hyun J.W. (2013). Diphlorethohydroxycarmalol attenuated cell damage against UVB radiation via enhancing antioxidant effects and absorbing UVB ray in human HaCaT keratinocytes. Environ. Toxicol. Pharmacol..

[12] Piao M.J., Susara Ruwan Kumara M.H., Kim K.C., Kang K.A., Kang H.K., Lee N.H., Hyun J.W. (2015). Diphlorethohydroxycarmalol suppresses ultraviolet B-Induced matrix metalloproteinases via inhibition of JNK and ERK signaling in human Keratinocytes. Biomol. Ther..

[13] Piao M.J., Ahn M.J., Kang K.A., Ryu Y.S., Hyun Y., Shilnikova K., Zhen A.X., Jeong J.W., Choi Y.H., Kang H.K. (2018). Particulate matter 2.5 damages skin cells by inducing oxidative stress, subcellular organelle dysfunction, and apoptosis. Arch. Toxicol..

[14] Pauly M., Angebault-Prouteau C., Dridi H., Notarnicola C., Scheuermann V., Lacampagne A., Matecki S., Fauconnier J. (2017). ER stress disturbs SR/ER-mitochondria Ca^2+^ transfer: Implications in Duchenne muscular dystrophy. Biochim. Biophys. Acta Mol. Basis Dis..

[15] Nishitoh H. (2012). CHOP is a multifunctional transcription factor in the ER stress response. J. Biochem..

[16] Szegezdi E., Logue S.E., Gorman A.M., Samali A. (2006). Mediators of endoplasmic reticulum stress-induced apoptosis. EMBO Rep..

[17] Lee W.S., Yoo W.H., Chae H.J. (2015). ER Stress and Autophagy. Curr. Mol. Med..

[18] Chaudhary A.K., Yadav N., Bhat T.A., O’Malley J., Kumar S., Chandra D. (2016). A potential role of X-linked inhibitor of apoptosis protein in mitochondrial membrane permeabilization and its implication in cancer therapy. Drug Discov. Today.

[19] Piao M.J., Hewage S.R., Han X., Kang K.A., Kang H.K., Lee N.H., Hyun J.W. (2015). Protective effect of diphlorethohydroxycarmalol against ultraviolet B radiation-induced DNA damage by inducing the nucleotide excision repair system in HaCaT human keratinocytes. Mar. Drugs.

[20] Zou Y., Qian Z.J., Li Y., Kim M.M., Lee S.H., Kim S.K. (2008). Antioxidant effects of phlorotannins isolated from Ishige okamurae in free radical mediated oxidative systems. J. Agric. Food Chem..

[21] Ahn M.J., Yoon K.D., Kim C.Y., Kim J.H., Shin C.G., Kim J. (2006). Inhibitory activity on HIV-1 reverse transcriptase and integrase of a carmalol derivative from a brown Alga, *Ishige* okamurae. Phytother. Res..

[22] Heo S.J., Hwang J.Y., Choi J.I., Han J.S., Kim H.J., Jeon Y.J. (2009). Diphlorethohydroxycarmalol isolated from Ishige okamurae, a brown algae, a potent alpha-glucosidase and alpha-amylase inhibitor, alleviates postprandial hyperglycemia in diabetic mice. Eur. J. Pharmacol..

[23] Heo S.J., Ko S.C., Kang S.M., Cha S.H., Lee S.H., Kang D.H., Jung W.K., Affan A., Oh C., Jeon Y.J. (2010). Inhibitory effect of diphlorethohydroxycarmalol on melanogenesis and its protective effect against UV-B radiation-induced cell damage. Food Chem. Toxicol..

[24] Ahn M., Moon C., Yang W., Ko E.J., Hyun J.W., Joo H.G., Jee Y., Lee N.H., Park J.W., Ko R.K. (2011). Diphlorethohydroxycarmalol, isolated from the brown algae *Ishige okamurae*, protects against radiation-induced cell damage in mice. Food Chem. Toxicol..

[25] Krutmann J., Liu W., Li L., Pan X., Crawford M., Sore G., Seite S. (2014). Pollution and skin: From epidemiological and mechanistic studies to clinical implications. J. Dermatol. Sci..

[26] Li Q., Kang Z., Jiang S., Zhao J., Yan S., Xu F., Xu J. (2017). Effects of ambient fine particles PM2.5 on human HaCaT cells. Int. J. Environ. Res. Public Health.

[27] Hu R., Xie X.Y., Xu S.K., Wang Y.N., Jiang M., Wen L.R., Lai W., Guan L. (2017). PM2.5 exposure elicits oxidative stress responses and mitochondrial apoptosis pathway activation in HaCaT keratinocytes. Chin. Med. J..

[28] Mei Y., Thompson M.D., Cohen R.A., Tong X. (2013). Endoplasmic reticulum stress and related pathological processes. J. Pharmacol. Biomed. Anal..

[29] Sano R., Reed J.C. (2013). ER stress-induced cell death mechanisms. Biochim. Biophys. Acta.

[30] Shrestha A., Pun N.T., Park P.H. (2018). ZFP36L1 and AUF1 induction contribute to the suppression of inflammatory mediators expression by globular adiponectin via autophagy induction in macrophages. Biomol. Ther..

[31] Ahn M.Y., Jee S.D., Hwang J.S., Yun E.Y., Ahn K.S., Kim Y.S. (2013). Anti-inflammatory effect of isaria sinclairii glycosaminoglycan in an adjuvant-treated arthritis rat model. Toxicol. Res..

[32] De Chiara G., Marcocci M.E., Torcia M., Lucibello M., Rosini P., Bonini P., Higashimoto Y., Damonte G., Armirotti A., Amodei S. (2006). Bcl-2 phosphorylation by p38 MAPK: Identification of target sites and biologic consequences. J. Biol. Chem..

[33] Kim B.J., Ryu S.W., Song B.J. (2006). JNK- and p38 kinase-mediated phosphorylation of Bax leads to its activation and mitochondrial translocation and to apoptosis of human hepatoma HepG2 cells. J. Biol. Chem..

[34] Lee S.J., Kim M.S., Park J.Y., Woo J.S., Kim Y.K. (2008). 15-Deoxy-delta 12,14-prostaglandin J2 induces apoptosis via JNK-mediated mitochondrial pathway in osteoblastic cells. Toxicology.

[35] Jung S., Lim J., Kwon S., Jeon S., Kim J., Lee J., Kim S. (2017). Characterization of particulate matter from diesel passenger cars tested on chassis dynamometers. J. Environ. Sci..

[36] Lee B.K., Smith T.J., Garshick E., Natkin J., Reaser P., Lane K., Lee H.K. (2005). Exposure of trucking company workers to particulate matter during the winter. Chemosphere.

[37] Park J.E., Piao M.J., Kang K.A., Shilnikova K., Hyun Y.J., Oh S.K., Jeong Y.J., Chae S., Hyun J.W. (2017). A benzylideneacetophenone derivative induces apoptosis of radiation-resistant human breast cancer cells via oxidative stress. Biomol. Ther..

[38] Piao M.J., Ahn M.J., Kang K.A., Kim K.C., Zheng J., Yao C.W., Cha J.W., Hyun C.L., Kang H.K., Lee N.H. (2014). Phloroglucinol inhibits ultraviolet B radiation-induced oxidative stress in the mouse skin. Int. J. Radiat. Biol..

[39] Li D., Li L., Li P., Li Y., Chen X. (2015). Apoptosis of HeLa cells induced by a new targeting photosensitizer-based PDT via a mitochondrial pathway and ER stress. Onco Targets Ther..

[40] Park J.E., Hyun Y.J., Piao M.J., Kang K.A., Ryu Y.S., Shilnikova K., Zhen A.X., Ahn M.J., Ahn Y.S., Koh Y.S. (2018). Mackerel-derived fermented fish oil protects skin against UVB-induced cellular damage by inhibiting oxidative stress. J. Funct. Foods.

